# Morphology and Multi-Gene Phylogeny Reveal *Pestalotiopsis pinicola* sp. nov. and a New Host Record of *Cladosporium anthropophilum* from Edible Pine (*Pinus armandii*) Seeds in Yunnan Province, China

**DOI:** 10.3390/pathogens8040285

**Published:** 2019-12-04

**Authors:** Saowaluck Tibpromma, Peter E. Mortimer, Samantha C. Karunarathna, Fangdong Zhan, Jianchu Xu, Itthayakorn Promputtha, Kai Yan

**Affiliations:** 1 College of Resources and Environment, Yunnan Agricultural University, Kunming 650201, Yunnan, China; saowaluckfai@gmail.com (S.T.); zfd97@ynau.edu.cn (F.Z.); 2 Key Laboratory for Plant Diversity and Biogeography of East Asia, Kunming Institute of Botany, Chinese Academy of Science, Kunming 650201, Yunnan, China; samanthakarunarathna@gmail.com (S.C.K.); jxu@mail.kib.ac.cn (J.X.); 3 Department of Biology, Faculty of Science, Chiang Mai University, Chiang Mai 50200, Thailand; ppam118@hotmail.com; 4 Center of Excellence in Bioresources for Agriculture, Industry and Medicine, Department of Biology, Faculty of Science, Chiang Mai University, Muang District, Chiang Mai 50200, Thailand

**Keywords:** ascomycota, endophytic fungi, new taxon, saprobic fungi, taxonomy, weak pathogen

## Abstract

This study contributes new knowledge on the diversity of conidial fungi in edible pine (*Pinus armandii*) seeds found in Yunnan Province, China and emphasizes the importance of edible seed products to ensure food safety standards. We isolated two fungal species, one on the pine seed coat and the other on the endosperm of the pine seed. The two fungal species were identified as *Pestalotiopsis pinicola* sp. nov. and a new host record *Cladosporium anthropophilum*. Characteristic morphological features of *Pestalotiopsis pinicola* were used alongside results from multi-gene phylogenetic analysis to distinguish it from currently known species within the genus. *Cladosporium anthropophilum* was identified as a new host record based on morphological features and phylogenetic analysis. In addition, detailed descriptions, scanned electron microscopy morphology, illustrations, and phylogenetic trees are provided to show the placement of these species.

## 1. Introduction 

Chinese white pine (*Pinus armandii*), one of the endemic conifer species of East Asia, is known throughout China, and particularly Yunnan Province, for its substantial ecological and economic value [1,2]. *Pinus armandii* seeds are suitable for use as a culinary ingredient after roasting, because the fatty acid profile of the seeds has a higher level of taxoleic acid and lower levels of octadecenoic acids compared to other species in *Pinus* [3,4]. 

Seeds are colonized by various types of fungi including fungal pathogens [5]. Several fungal species exist in seeds in the forms of spores and mycelium and can subsist for long periods of time on the seed coat and in the inner areas [6]. In general, fungi that are present within seeds are more harmful than those that merely contaminate the outer seed coat [6]. Common fungi genera that have been reported as associated with various seeds are *Aspergillus*, *Mucor*, *Penicillium*, *Pestalotiopsis*, *Rhizopus*, and *Trichoderma* [7]. Some studies have confirmed that fungi that are usually thought to be saprobes act as pathogens under certain circumstances, while endophytes can also switch to a saprobic lifestyle [8,9]. Fungal invasions happen after injury to the seed or seed coat as well as when moisture levels and temperatures are favorable for fungal growth [10]. Many seed fungi are also important sources of bioactive compounds [11,12]. In this study, we were able to isolate and identify two micro-fungi belonging to the genera *Cladosporium* and *Pestalotiopsis* from the seeds of *Pinus armandii*.

The genus *Cladosporium* (Cladosporiaceae, Capnodiales) was introduced by Link [13] with *C. herbarum* (Pers.) Link as the type species. The members of this genus can be endophytes, pathogens, and saprobes with worldwide distribution across a wide range of disparate substrates [14,15,16,17,18]. *Cladosporium* species are also known as the most abundant fungi in indoor and outdoor environments and are also important as spoilage organisms and discoloration which have been screened from cereal grains, fruits, peanuts, and chilled meat [19,20,21,22]. While *Cladosporium* species have not been reported as mycotoxin producers, they may nonetheless represent a health threat. Furthermore, some species have been reported causing fungal allergies, especially in patients with severe asthma [23,24,25,26,27,28,29,30,31]. Recently, several *Cladosporium* species have been reported in China, Thailand, and the United Kingdom on the decaying seed pods of *Delonix regia, Entada phaseoloides, Laburnum anagyroides*, and *Magnolia grandiflora* [32]. Only two species, *Cladosporium nigrellum* and *C. psoraleae*, have been reported from *Pinus armandii* in China [33,34]. 

The genus *Pestalotiopsis* (Sporocadaceae, Amphisphaeriales) was introduced by Steyaert [35] with *P. guepinii* (Desm.) Steyaert as the type species. The members of this genus can be found worldwide as endophytes, saprobes or opportunistic pathogens [18,36,37,38,39,40,41,42,43,44]. Some of them are confirmed to cause human and animal diseases [42,45,46]. For example, *Pestalotiopsis* spp. have been isolated from a bronchial biopsy, corneal abrasions, eyes, feet, fingernails, scalp, and sinuses from the human body [45]. In addition, this genus is known as one of the common fungi genera reported on various seeds [7]. *Pestalotiopsis algeriensis*, *P. carveri*, *P. caudata*, *P. cocculi*, *P. disseminate*, *P. heterocornis*, *P. lespedezae*, *P. neglecta*, *P. olivacea*, and *P. vismiae* have been reported from *Pinus armandii* in China [33,34,38,47]. 

In the present study, we used multi-gene sequence analysis, morphological examinations, and culture characteristics for the identification and delimitation of fungi isolates belonging to the genera *Cladosporium* and *Pestalotiopsis* from seeds of *Pinus armandii* collected in Yunnan Province, China.

## 2. Materials and Methods

### 2.1. Sample Collection and Specimen Examination 

**Figure 1:**
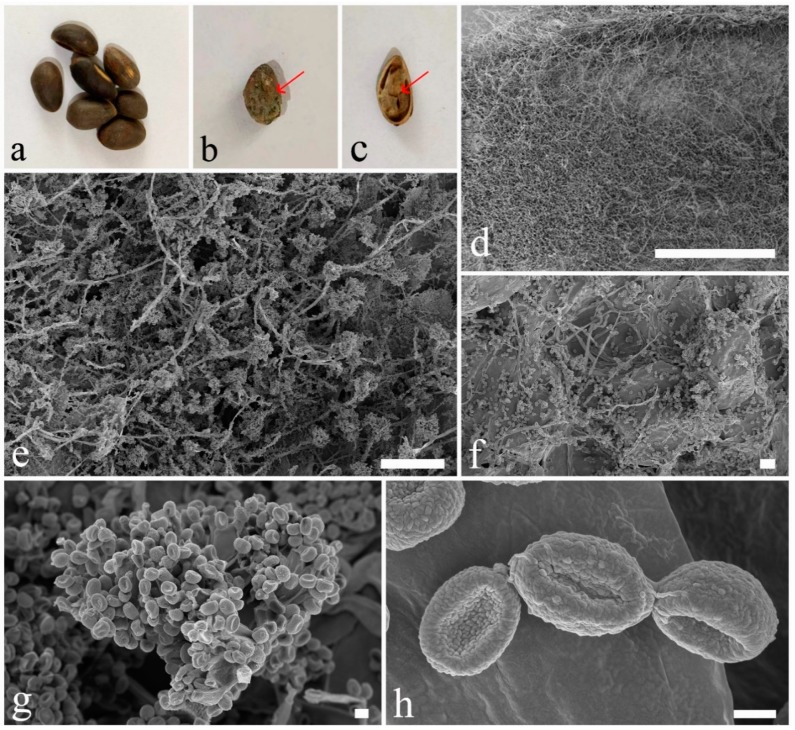
(**a**) Seeds of *Pinus armandii* from Yunnan Province, China. (**b**) Fungal mass on a seed. (**c**) Endosperm covered with mycelia. (**d**) Mycelia on endosperm under a SEM micrograph. (**e**–**h**) Rows of rounded cells present on a seed coat skin under SEM micrographs that form aerial hyphae, conidia, and conidiophores. Scale bars: d = 1 mm, e = 100 µm, f = 10 µm, g = 2 µm, h = 1 µm.

Fresh fungal structures (mycelia and spore masses) were directly isolated in potato dextrose agar (PDA) from seed coats and endosperms of *Pinus armandii* seeds using aseptic techniques, and the PDA plates were incubated at room temperature. Pine seeds were obtained outside Kunming, Yunnan Province, China (Figure 1). The seeds were then carefully analyzed. Morphological structures of the fungi were examined under a stereo microscope. Scanning electron microscopy (SEM) micrographs were obtained under a ZEISS GeminiSEM and ZEISS Sigma 300 apparatus, following the methods described by Figueras and Guarro [48]. To observe the fungal structures, sporulated cultures were mounted on water. Microscopic fungal structures were observed under a compound microscope and photographs were captured with a digital camera fitted on to the microscope. All microscopic structures of fungi were measured by the Tarosoft Image Framework program v.0.9.0.7., and Adobe Photoshop CS3 Extended version 10.0 (Adobe Systems, USA) was used to process and edit the images used in the figures.

#### Isolation

The PDA medium was used for culturing the isolated fungi. Spore masses from the seed coat and mycelia from the endosperm were aseptically transferred to PDA plates (two isolates of each species). The pure culture plates were incubated at room temperature (20–25 °C) for 14–21 days, and the fungal colonies were carefully observed and described. The herbarium specimens of the fungi were dehydrated using silica gel and deposited in the Mae Fah Luang University Herbarium. The pure cultures were deposited in the Kunming Institute of Botany Culture Collection (KMUCC). Index Fungorum (IF) and Facesoffungi (FoF) numbers were obtained as described by Index Fungorum [49] and Jayasiri et al. [50].

### 2.2. DNA Extraction, PCR Amplification, and DNA Sequencing

The mycelia of the cultures grown on PDA at room temperature for 4 weeks were used for DNA extraction. The fungal mycelia were scraped off with a sterile scalpel and transferred to 1.5 mL micro-centrifuge tubes under aseptic conditions and kept at −20 °C to avoid contaminations until use. The Biospin Fungal Genomic DNA Extraction Kit (BioFlux, China) was used to perform DNA extraction from the fungal cultures, following the manufacturer’s protocols. To amplify partial gene regions of the 5.8S rRNA gene in the internal transcribed spacer (*ITS*), translation elongation factor 1-alpha gene (*TEF1*), actin gene (*ACT*), and beta-tubulin gene (*TUB2*), polymerase chain reaction (PCR) was used. The PCR conditions and primers were set under standard conditions as shown in Table 1. The total volume of PCR mixtures for amplifications was set as described in Tibpromma et al. [18]. Purification and sequencing of PCR products were done by Sangon Biotech Co., Shanghai, China. 

### 2.3. Phylogenetic Analyses

The *ITS* and *TEF1* sequence data produced in this study were used in BLAST searches in the GenBank database (www.http://blast.ncbi.nlm.nih.gov/) to determine their most probable closely related taxa. The sequence data generated in this study were analyzed with closely related taxa retrieved from GenBank based on BLAST searches and recent publications [9,16,18,55,56]. Single gene sequence datasets were aligned using the MAFFT v.7.215 website [57] and manually edited in BioEdit v.7.0 when necessary [58]. Single sequence alignment datasets were combined using BioEdit v.7.2.5 [58]. The alignment of combined datasets in FASTA format was converted to PHYLIP and NEXUS formats using the Alignment Transformation Environment (ALTER) website [59]. Phylogenetic trees were run in randomized accelerated maximum likelihood (RAxML) and Bayesian posterior probabilities (BYPPs). The maximum likelihood (ML) analysis was performed via the CIPRES Science Gateway [60] using the RAxML-HPC BlackBox (8.2.4) section [61,62] with the general time reversible model (GTR) using a discrete gamma distribution as the evolutionary model. To carry out Bayesian analysis, the model of evolution was estimated using MrModeltest 2.2 [63] with HKY+I+G (for the *Pestalotiopsis* dataset) and GTR+I+G (for the *Cladosporium* dataset) as nucleotide substitution models selected for combined datasets. Posterior probabilities (PPs) [64] were determined by Markov chain Monte Carlo sampling (MCMC) in MrBayes v.3.0b4 [65]. The parameters were set as six simultaneous Markov chains ran for 5,000,000 generations and sampling every 100th generation for a total of 50,000 trees [66]. The first trees representing the burn-in phase of the analysis (20%) were discarded and the remaining (post-burn) trees were used for calculating PPs in the majority rule consensus tree (the critical value for the topological convergence diagnostic values reached 0.01) [67,68]. 

The phylograms were figured in FigTree v.1.4 [69] and reorganized using Microsoft Office PowerPoint 2007 and Adobe illustrator CS3 (Adobe Systems Inc., USA). The sequences generated in this study were submitted to GenBank (Table 2 and Table 3).

## 3. Results 

### 3.1. Phylogenetic Analysis of Combined Sequence Data

**Figure 2:**
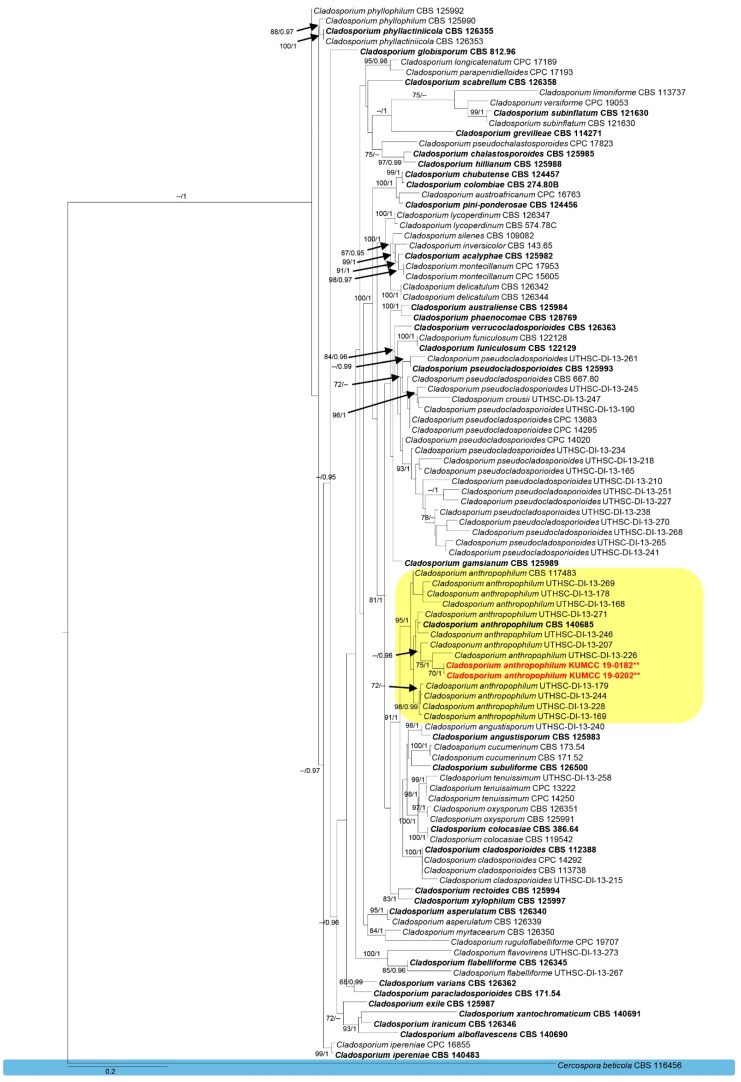
Phylogram generated from RAxML analysis based on combined *TEF1*, *ACT* and *ITS* sequence data of the genus *Cladosporium*. Related sequences were obtained from Sandoval-Denis et al. [16] and Bensch et al. [56]. Bootstrap support values for ML equal to or greater than 60% and BYPP from MCMC analyses equal to or greater than 0.95 are given above/below the nodes. The ex-type strains are indicated in bold type. Newly generated sequences are indicated in red with two asterisks.

The combined dataset of genera *Cladosporium* and *Pestalotiopsis* were analyzed using maximum likelihood and Bayesian analyses (Figure 2; Figure 4). Both the ML and BYPP trees showed similar results in topology and no significant differences were seen (data not presented). 

In the *Cladosporium* tree (Figure 2), the final alignments contained 104 strains with 1484 characters, including 594 characters for *TEF1*, 306 characters for *ACT*, and 584 characters for *ITS*. *Cercospora beticola* (CBS 116456) was used as an outgroup taxon. The tree topology of the ML analysis was similar to the BYPP. The best scoring RAxML tree with a final likelihood value of −14,457.527098 is presented. The matrix had 681 distinct alignment patterns with 30.20% undetermined characters or gaps. Estimated base frequencies were as follows: A = 0.228336, C = 0.290122, G = 0.251877, T = 0.229664; substitution rates AC = 1.724785, AG = 2.866615, AT = 1.692026, CG = 1.001444, CT = 5.300862, GT = 1.000000; gamma distribution shape parameter a = 0.312597. The phylogram of the genus *Cladosporium* based on a combined dataset showed that our strains grouped together with *Cladosporium anthropophilum* clade with relatively high bootstrap supports (Figure 2). 

In the *Pestalotiopsis* tree (Figure 4), the final alignments contained 81 strains with 1562 characters, including 549 characters for *TEF1*, 440 characters for *TUB2*, and 573 characters for *ITS*. *Neopestalotiopsis formicarum* (CBS 362.72) and *N. clavispora* (CBS 447.73) were used as outgroup taxa. The tree topology of the ML analysis was similar to the BYPP. The best scoring RAxML tree with a final likelihood value of −11413.131729 is presented. The matrix had 696 distinct alignment patterns, with 12.40% undetermined characters or gaps. Estimated base frequencies were as follows: A = 0.235816, C = 0.293897, G = 0.211788, T = 0.258500; substitution rates AC = 1.049115, AG = 3.327441, AT = 1.067008, CG = 0.861291, CT = 3.485808, GT = 1.000000; gamma distribution shape parameter a = 0.276615. The *Pestalotiopsis* phylogram, based on a combined dataset, showed that our new species, *Pestalotiopsis pinicola*, was well separated from *P. rosea* with relatively high bootstrap supports (100% ML/ 1 BYPP, Figure 4). Therefore, we propose *Pestalotiopsis pinicola* as a distinct new species and *Cladosporium anthropophilum* as a previously known species.

### 3.2. Taxonomy 

*Cladosporium anthropophilum* Sand.-Den., Gené and Wiederhold, Persoonia 36: 290 (2016) [16]. 

**Figure 3:**
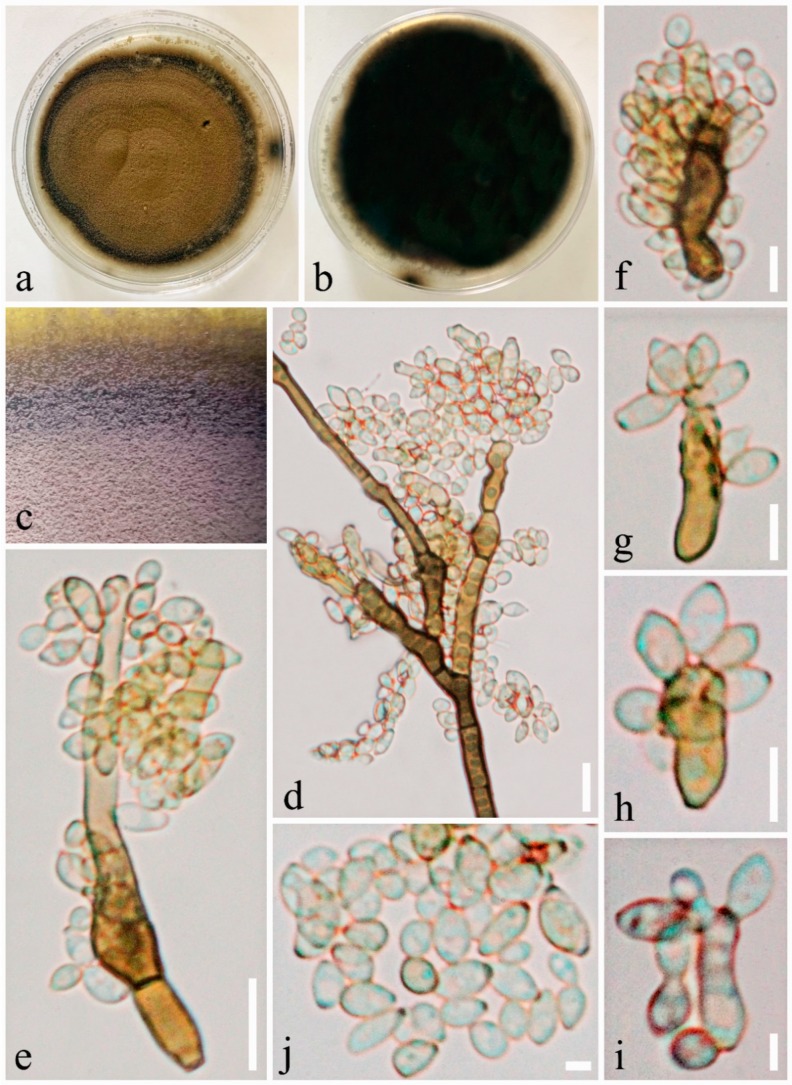
*Cladosporium anthropophilum* (KUMCC 19-0182). (**a**,**b**) Colony on PDA media. (**c**) Mycelium masses. (**d**,**e**) Conidiophores and conidiogenous cells and conidia. (**f**–**i**) Conidiogenous cells with secondary ramoconidia and coidia. (**j**) Conidia. Scale bars: (**d**,**e**) = 10 µm, (**f**–**i**) = 5 µm, (**j**) = 2 µm.

Index Fungorum number: IF815334, Facesoffungi number: FoF 06275, Figure 3.

*Saprobic or weak pathogen* on seed coat of *Pinus armandii*. Sexual morph: Undetermined. Asexual morph: Mycelium sparsely formed, superficial, overgrowing entire pod, thin to dense, later often forming colonies on the surface, hyphae straight to strongly flexuous-sinuous, branched, subhyaline to olivaceous-brown. *Conidiophores* erect, stipes, slightly attenuated towards the apex, yellow-brown to dark-brown, smooth and thick-walled, branched, septate. *Conidiogenous cells* 5–15 × 2.5–5.5 μm ($\bar{x}$ = 8.7 × 4 μm; *n* = 20), cylindrical, sometimes geniculate-sinuous, proliferation sympodia with distinctive scar. *Secondary ramoconidia* 5.9–9.1 × 2–3.5 μm ($\bar{x}$ = 7.7 × 2.9 μm; *n* = 40), olivaceous-brown, ellipsoid-ovoid, obovoid, fusiform, subcylindrical, aseptate, smooth to rough-walled, granulate and scars. *Conidia* 2.7–5.6 × 2–3.2 μm ($\bar{x}$ = 4.1 × 2.7 μm; *n* = 40), in simple or branched chains, subhyaline to olivaceous, ellipsoid-ovoid, aseptate, a scar at base, rough-walled with granulate.

*Culture characters:* Colonies on PDA reaching 9 cm in diameter after 3 weeks at room temperature. Colonies olivaceous-grey to olivaceous, pale-olivaceous to black at the margin and circular with slightly regular colony, powdery, radially furrowed, aerial mycelium sparse with raised elevation, numerous small prominent exudates formed, sporulation profuse.

*Material examined*: CHINA, Yunnan Province, on seed coat of *Pinus armandii* Franch., May 2019, Kai Yan, Seed01 (MFLU19-2362); living culture KUMCC 19-0182 = KUMCC 19-0202.

**Figure 4:**
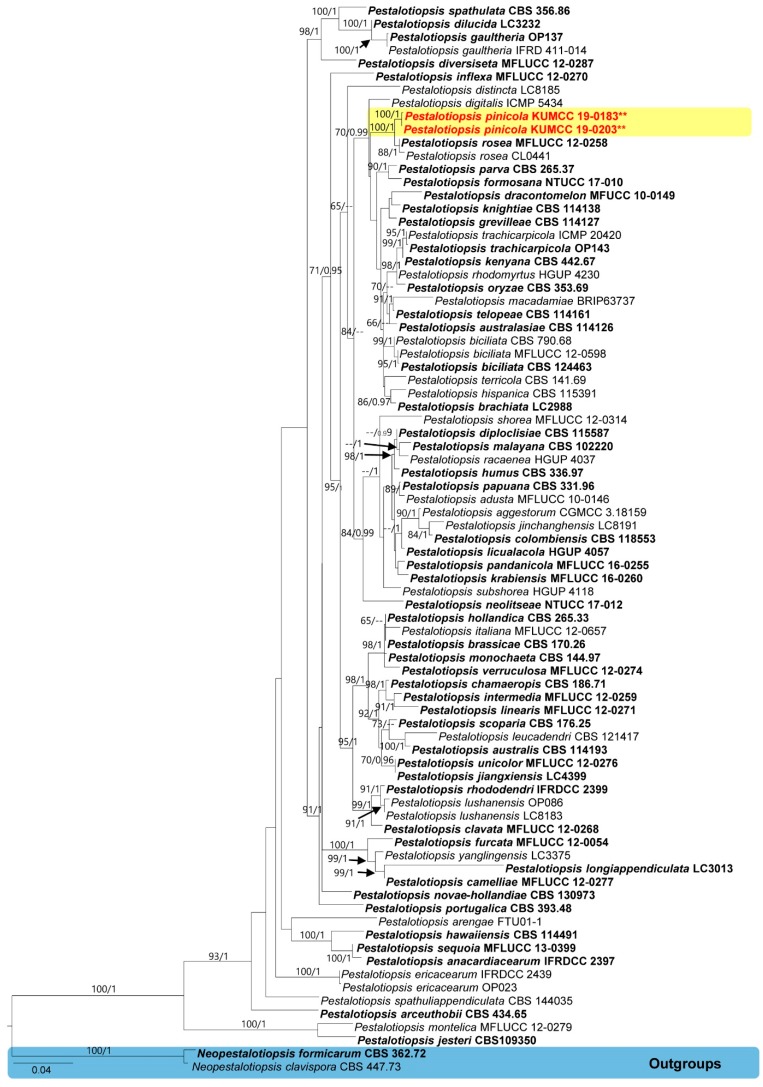
Phylogram generated from RAxML analysis based on combined *TEF1*, *TUB2*, and *ITS* sequence data of the genus *Pestalotiopsis*. Related sequences were obtained from Ariyawansa et al. [55] and Tibpromma et al. [9,18]. Bootstrap support values for ML equal to or greater than 60% and BYPP from MCMC analyses equal to or greater than 0.95 are given above/below the nodes. The ex-type strains are indicated in bold type. Newly generated sequences are indicated in red with two asterisks (**).

Note that *Cladosporium anthropophilum* was established by Sandoval-Denis et al. [16] which belongs to the *C. cladosporioides* species complex. *Cladosporium anthropophilum* is probably known as a common saprobic fungus and also represents a clinically relevant fungus [16,70]. In this study, we found a strain of *C. anthropophilum* from a seed coat of *Pinus armandii* which was confirmed based on morphology and multi-gene analysis (Figure 2 and Figure 3). The morphology of our strain was similar to the *C. anthropophilum* described by Sandoval-Denis et al. [16]. In addition, this is the first report of *C. anthropophilum* from *P. armandii* (Figure 4).

***Pestalotiopsis pinicola*** Tibpromma, Karunaratha and Mortimer, *sp. nov.*

**Figure 5:**
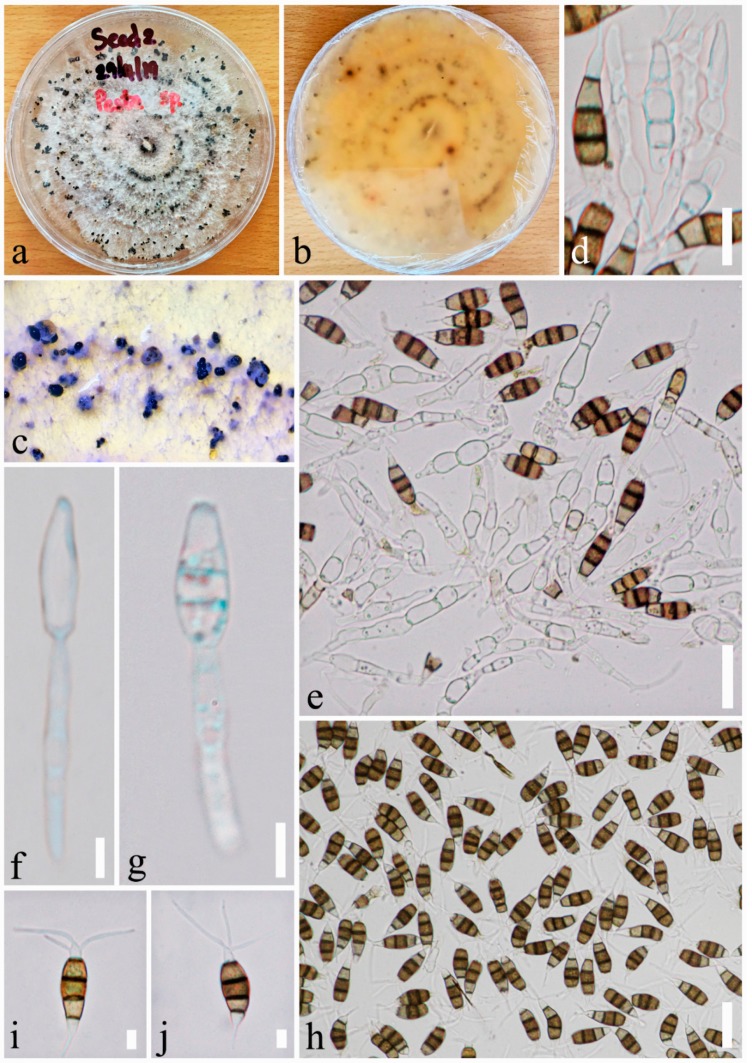
*Pestalotiopsis pinicola* (KUMCC 19-0183, ex-type). (**a**,**b**) Colony on PDA media. (**c**) Fruiting body on PDA media. (**d**–**g**) Conidia, conidiogenous cells and conidia. (**h**–**j**) Conidia. Scale bars: (**d**) = 5 µm, (**e**) = 10 µm, (**f**,**g**) = 5 µm, (**h**) = 20 µm, (**i**–**j**) = 5 µm.

Index Fungorum number: IF556765, Facesoffungi number: FoF 06276, Figure 5.
*Etymology*: named after the host genus, *Pinus*.
*Holotype*: MFLU19-2363.

*Saprobic or endophytic* on seed endosperm of *Pinus armandii*. Sexual morph: Undetermined. Asexual morph: *Conidiophores* short, unbranched, reduced to conidiogenous cells. *Conidiogenous cells* discrete, holoblastic, simple, filiform, smooth and thin-walled, hyaline. *Conidia* fusoid to ellipsoid, straight to slightly curved, 3–4 septate (mostly 4 septate), 18–23 × 5–7 μm ($\bar{x}$ = 21 × 6 μm, *n* = 40), basal cell conic to obconic with obtuse end, subhyaline, thin-walled, verruculose, 3.5–5 μm long ($\bar{x}$ = 4 μm); three median cells, doliiform, yellow-brown and becoming brown with age, septa and periclinal walls darker than rest of the cell, together 11–16 μm long ($\bar{x}$ = 13 μm); second cell from base 3–6 μm long ($\bar{x}$ = 4.5 μm); third cell 3–5.5 μm long ($\bar{x}$ = 4.6 μm); fourth cell 3–5 μm long ($\bar{x}$ = 3.9 μm); apical cell hyaline, conic 3–5 μm long ($\bar{x}$ = 3.9 μm), with 2(–3) tubular apical appendages; appendages arising from the apex of the apical cell, unbranched, 5–17 μm long ($\bar{x}$ = 10.3 μm); single basal appendage usually present, 2–7 μm long ($\bar{x}$ = 4.7 μm), tubular, unbranched, centric. 

*Culture characteristics*: Colonies on PDA reaching 9 cm in diameter after 2 weeks at room temperature, edge undulate with curled, whitish, aerial mycelium on surface, spore masses form after 1 month, black spore masses; reverse of culture yellow-white to yellow-orange with black dots.

*Material examined:* China, Yunnan Province, on endosperm of pine seed of *Pinus armandii* Franch., May 2019, Kai Yan, Seed02 (MFLU19-2363, holotype); ex-type living culture KUMCC 19-0183 = KUMCC 19-0203.

Note that *Pestalotiopsis pinicola* is introduced based on morphological and phylogenetic data. In the phylogenetic analysis, our new species cluster with *P. rosea* Maharachch. and K.D. Hyde [40] but are well separated with high support (100% ML/1 BYPP, Figure 4). In addition, base pair differences of our new taxa with closest taxa were checked based on the recommendations of Jeewon and Hyde [71]; our isolate differs from *P. rosea* (MFLUCC12-0258 and CL0441) with five *ITS* base pairs (2.65%), four *TUB* base pairs (1.64%), and ten *RPB2* base pairs (4.65%). In addition, the culture of *P. rosea* was seen as a reddish colony [40], while our new species produces a whitish colony.

In a BLASTn search on the NCBI GenBank, the closest *ITS* sequence match of KUMCC 19-0183 is *Pestalotiopsis* sp. with a 99.31% identity to the strain JSM 06261592 (KY086253), KUMCC 19-0203 is *P. neglecta* with 99.82% identity to the strain CBS 357.71 (MH860161.1), the closest *TEF1* sequence matches of KUMCC 19-0183 and KUMCC 19-0203 were with the *P. rosea* strain MFLUCC12-0258 with 98.72% (JX399069), while the closest matches with the *TUB2* sequence were with the 99.53% identical *P. olivacea* strain PSHI2002 (DQ787834) by KUMCC 19-0183 and 99.53% identical *P. vismiae* strain Q15DY (EF055259) by KUMCC 19-0203. 

## 4. Discussion

In this paper, we describe a novel taxon belonging to *Pestalotiopsis* and a new host record of *Cladosporium* isolated from seeds of *Pinus armandii* obtained from Yunnan Province, China. Mature agar colonies sporulated in cultures with masses of conidia. 

We isolated a new *Pestalotiopsis* species from mycelia-covered endosperms of pine seeds. Past research has yielded new species from *Pestalotiopsis* with similar origins; for example, several endophytic *Pestalotiopsis* species were isolated from the bark and needles of *Pinus armandii* in China [38]. Furthermore, *Pestalotiopsis brassicae* and *P. oryzae* were isolated from seeds from *Oryza sativa* and *Brassica napus* [42]. Several have often been isolated as endophytes and many pathogens or endophytes may persist as saprobes, which mean *Pestalotiopsis* species are able to switch life-modes [42]. The present study illustrates a novel species of *Pestalotiopsis* as *Pestalotiopsis pinicola*, taking both morphology and phylogeny into consideration (Figure 4 and Figure 5). The phylogenetic tree construction of the DNA sequences of single and combined genes provides confirmation with high bootstrap support that *P. pinicola* is a characteristic new species separate from other known species of the genus (Figure 4). Moreover, this genus is known as one of the fungal groups that can produce a wide range of chemically novel secondary metabolites and mycotoxins; for example, pestaloside exhibiting significant antifungal properties was produced by *P. microspora*, obtained from *Torreya taxifolia* [42,72,73,74]. There is, consequently, a potential health threat in the sale of these seeds as an edible foodstuff. Follow-up research investigating the potential toxins produced by *P. pinicola* should be conducted to clarify this issue. We conclude that fungi live inside seeds as endophytes and then switch life-modes to saprobes or weak pathogens when conditions become unfavorable. In the future, knowledge about pestalotioid fungi associated with seeds will help provide a basis for developing proper management of these pathogens. 

We found another species, *Cladosporium anthropophilum*, growing on pine seed coats. The etymology of this species comes from Greek which refers to the sample’s source which was isolated from a human clinical sample [16]. This species can be found in human clinical samples, indoor air, food and plant materials, such as seeds or leaves, and it is also a common saprobic fungus [56]. In addition, this species is known as the second-most prevalent species from clinical environments from the US after *C. halotolerans*, and it also has been isolated quite frequently from indoor environments [16,69]. However, we continue to lack information about the chemistry or secondary metabolites of this species along with the potential serious health effects associated with long-term exposure to a large amount of *Cladosporium anthropophilum*.

The present study illustrates two species of *Pestalotiopsis* and *Cladosporium* based on both morphology and phylogeny. These two species of fungi were isolated from pine seeds from Yunnan Province, China. The fungal mycelia in the seeds were observed after the seeds were broken open to eat, and these seeds can be found in many food markets around Yunnan Province. We recommend that consumers should carefully check seed products before purchase and consumption, as these fungi may cause adverse health effects in the long term. Therefore, to address this health concern, in the future we will focus our research on the secondary metabolites and mycotoxins of *Cladosporium anthropophilum* and *Pestalotiopsis pinicola.*

## Figures and Tables

**Table 1:** Gene regions and primers used in this study.

Genes	Primers (Forward/Reverse)	References
***Cladosporium***		
*ACT*	512F/783R	[51]
*ITS*	ITS5/ITS4	[52]
*TEF1*	728F/986R	[51]
***Pestalotiopsis***		
*ITS*	ITS5/ITS4	[52]
*TEF1*	526F/1567R	[53]
*TUB2*	T1/T2	[54]

**Table 2:** GenBank accession numbers and culture collection numbers of the nucleotide sequences of *Cladosporium* taxa used in this study. The new sequences generated in this study are in bold type.

Species	Culture Collection Number	GenBank Accession Numbers		
		*ITS*	*TEF1*	*ACT*
*Cercospora beticola*	CBS 116456	NR_121315	AY840494	AY840458
*Cladosporium acalyphae*	CBS 125982	HM147994	HM148235	HM148481
*C. alboflavescens*	CBS 140690	LN834420	LN834516	LN834604
*C. angustisporum*	CBS 125983	HM147995	HM148236	HM148482
*C. angustisporum*	UTHSC-DI-13-240	LN834356	LN834452	LN834540
*C. anthropophilum*	CBS 117483	HM148007	HM148248	HM148494
*C. anthropophilum*	CBS 140685	LN834437	LN834533	LN834621
*C. anthropophilum*	KUMCC 19-0182	MN412638	MN417513	MN417511
*C. anthropophilum*	KUMCC 19-0202	MN412639	MN417514	MN417512
*C. anthropophilum*	UTHSC-DI-13-168	LN834407	LN834503	LN834591
*C. anthropophilum*	UTHSC-DI-13-169	LN834408	LN834504	LN834592
*C. anthropophilum*	UTHSC-DI-13-178	LN834410	LN834506	LN834594
*C. anthropophilum*	UTHSC-DI-13-179	LN834411	LN834507	LN834595
*C. anthropophilum*	UTHSC-DI-13-207	LN834413	LN834509	LN834597
*C. anthropophilum*	UTHSC-DI-13-226	LN834421	LN834517	LN834605
*C. anthropophilum*	UTHSC-DI-13-228	LN834423	LN834519	LN834607
*C. anthropophilum*	UTHSC-DI-13-244	LN834428	LN834524	LN834612
*C. anthropophilum*	UTHSC-DI-13-246	LN834430	LN834526	LN834614
*C. anthropophilum*	UTHSC-DI-13-269	LN834437	LN834533	LN834621
*C. anthropophilum*	UTHSC-DI-13-271	LN834439	LN834535	LN834623
*C. asperulatum*	CBS 126339	HM147997	HM148238	HM148484
*C. asperulatum*	CBS 126340	HM147998	HM148239	HM148485
*C. australiense*	CBS 125984	HM147999	HM148240	HM148486
*C. austroafricanum*	CPC 16763	KT600381	KT600478	KT600577
*C. chalastosporoides*	CBS 125985	HM148001	HM148242	HM148488
*C. chubutense*	CBS 124457	FJ936158	FJ936161	FJ936165
*C. cladosporioides*	CBS 112388	HM148003	HM148244	HM148490
*C. cladosporioides*	CBS 113738	HM148004	HM148245	HM148491
*C. cladosporioides*	CPC 14292	HM148046	HM148287	HM148533
*C. cladosporioides*	UTHSC-DI-13-215	LN834360	LN834456	LN834544
*C. colocasiae*	CBS 119542	HM148066	HM148309	HM148554
*C. colocasiae*	CBS 386.64	HM148067	HM148310	HM148555
*C. colombiae*	CBS 274.80B	FJ936159	FJ936163	FJ936166
*C. crousii*	UTHSC-DI-13-247	LN834431	LN834527	LN834615
*C. cucumerinum*	CBS 171.52	HM148072	HM148316	HM148561
*C. cucumerinum*	CBS 173.54	HM148074	HM148318	HM148563
*C. delicatulum*	CBS 126342	HM148079	HM148323	HM148568
*C. delicatulum*	CBS 126344	HM148081	HM148325	HM148570
*C. exile*	CBS 125987	HM148091	HM148335	HM148580
*C. flabelliforme*	CBS 126345	HM148092	HM148336	HM148581
*C. flabelliforme*	UTHSC-DI-13-267	LN834361	LN834457	LN834545
*C. flavovirens*	UTHSC-DI-13-273	LN834440	LN834536	LN834624
*C. funiculosum*	CBS 122128	HM148093	HM148337	HM148582
*C. funiculosum*	CBS 122129	HM148094	HM148338	HM148583
*C. gamsianum*	CBS 125989	HM148095	HM148339	HM148584
*C. globisporum*	CBS 812.96	HM148096	HM148340	HM148585
*C. grevilleae*	CBS 114271	JF770450	JF770472	JF770473
*C. hillianum*	CBS 125988	HM148097	HM148341	HM148586
*C. inversicolor*	CBS 143.65	HM148100	HM148344	HM148589
*C. ipereniae*	CBS 140483	KT600394	KT600491	KT600589
*C. ipereniae*	CPC 16855	KT600395	KT600492	KT600590
*C. iranicum*	CBS 126346	HM148110	HM148354	HM148599
*C. limoniforme*	CBS 113737	KT600396	KT600493	KT600591
*C. longicatenatum*	CPC 17189	KT600403	KT600500	KT600598
*C. lycoperdinum*	CBS 126347	HM148112	HM148356	HM148601
*C. lycoperdinum*	CBS 574.78C	HM148115	HM148359	HM148604
*C. montecillanum*	CPC 15605	KT600407	KT600505	KT600603
*C. montecillanum*	CPC 17953	KT600406	KT600504	KT600602
*C. myrtacearum*	CBS 126350	HM148117	HM148361	HM148606
*C. oxysporum*	CBS 125991	HM148118	HM148362	HM148607
*C. oxysporum*	CBS 126351	HM148119	HM148363	HM148608
*C. paracladosporioides*	CBS 171.54	HM148120	HM148364	HM148609
*C. parapenidielloides*	CPC 17193	KT600410	KT600508	KT600606
*C. phaenocomae*	CBS 128769	JF499837	JF499875	JF499881
*C. phyllactiniicola*	CBS 126353	HM148151	HM148395	HM148640
*C. phyllactiniicola*	CBS 126355	HM148153	HM148397	HM148642
*C. phyllophilum*	CBS 125992	HM148154	HM148398	HM148643
*C. phyllophilum*	CBS 125990	HM148111	HM148355	HM148600
*C. pini-ponderosae*	CBS 124456	FJ936160	FJ936164	FJ936167
*C. pseudochalastosporoides*	CPC 17823	KT600415	KT600513	KT600611
*C. pseudocladosporioides*	CBS 125993	HM148158	HM148402	HM148647
*C. pseudocladosporioides*	CBS 667.80	HM148165	HM148409	HM148654
*C. pseudocladosporioides*	CPC 13683	HM148173	HM148417	HM148662
*C. pseudocladosporioides*	CPC 14020	HM148185	HM148429	HM148674
*C. pseudocladosporioides*	CPC 14295	HM148188	HM148432	HM148677
*C. pseudocladosporioides*	UTHSC-DI-13-165	LN834406	LN834502	LN834590
*C. pseudocladosporioides*	UTHSC-DI-13-190	LN834412	LN834508	LN834596
*C. pseudocladosporioides*	UTHSC-DI-13-210	LN834414	LN834510	LN834598
*C. pseudocladosporioides*	UTHSC-DI-13-218	LN834418	LN834514	LN834602
*C. pseudocladosporioides*	UTHSC-DI-13-227	LN834422	LN834518	LN834606
*C. pseudocladosporioides*	UTHSC-DI-13-234	LN834424	LN834520	LN834608
*C. pseudocladosporioides*	UTHSC-DI-13-238	LN834426	LN834522	LN834610
*C. pseudocladosporioides*	UTHSC-DI-13-241	LN834427	LN834523	LN834611
*C. pseudocladosporioides*	UTHSC-DI-13-245	LN834429	LN834525	LN834613
*C. pseudocladosporioides*	UTHSC-DI-13-251	LN834432	LN834528	LN834616
*C. pseudocladosporioides*	UTHSC-DI-13-261	LN834384	LN834480	LN834568
*C. pseudocladosporioides*	UTHSC-DI-13-265	LN834435	LN834531	LN834619
*C. pseudocladosporioides*	UTHSC-DI-13-268	LN834436	LN834532	LN834620
*C. pseudocladosporioides*	UTHSC-DI-13-270	LN834438	LN834534	LN834622
*C. rectoides*	CBS 125994	HM148193	HM148438	HM148683
*C. ruguloflabelliforme*	CPC 19707	KT600458	KT600557	KT600655
*C. scabrellum*	CBS 126358	HM148195	HM148440	HM148685
*C. silenes*	CBS 109082	EF679354	EF679429	EF679506
*C. subinflatum*	CBS 121630	EF679389	EF679467	EF679543
*C. subinflatum*	CBS 121630	EF679389	EF679467	EF679543
*C. subuliforme*	CBS 126500	HM148196	HM148441	HM148686
*C. tenuissimum*	CPC 13222	HM148210	HM148455	HM148700
*C. tenuissimum*	CPC 14250	HM148211	HM148456	HM148701
*C. tenuissimum*	UTHSC-DI-13-258	LN834404	LN834500	LN834588
*C. varians*	CBS 126362	HM148224	HM148470	HM148715
*C. verrucocladosporioides*	CBS 126363	HM148226	HM148472	HM148717
*C. versiforme*	CPC 19053	KT600417	KT600515	KT600613
*C. xantochromaticum*	CBS 140691	LN834415	LN834511	LN834599
*C. xylophilum*	CBS 125997	HM148230	HM148476	HM148721

**Table 3:** GenBank accession numbers and culture collection numbers of the nucleotide sequences of the *Pestalotiopsis* taxa used in this study. The new sequences generated in this study are in black bold type.

Species	Culture Collection Number	GenBank Accession Numbers		
		*ITS*	*TUB2*	*TEF1*
*Neopestalotiopsis clavispora*	CBS 447.73	KM199374	KM199443	KM199539
*N. formicarum*	CBS 362.72	KM199358	KM199455	KM199517
*Pestalotiopsis adusta*	MFLUCC 10-0146	JX399006	JX399037	JX399070
*P. aggestorum*	LC8186	KY464140	KY464160	KY464150
*P. anacardiacearum*	IFRDCC 2397	KC247154	KC247155	KC247156
*P. arceuthobii*	CBS 434.65	KM199341	KM199427	KM199516
*P. arengae*	CBS 331.92	KM199340	KM199426	KM199515
*P. australasiae*	CBS 114126	KM199297	KM199409	KM199499
*P. australis*	CBS 114193	KM199332	KM199383	KM199475
*P. biciliata*	CBS 124463	KM199308	KM199399	KM199505
*P. biciliata*	CBS 790.68	KM199305	KM199400	KM199507
*P. biciliata*	MFLUCC 12-0598	KX816920	KX816948	KX816890
*P. brachiata*	LC2988	KX894933	KX895265	KX895150
*P. brassicae*	CBS 170.26	KM199379	-	KM199558
*P. camelliae*	MFLUCC 12-0277	JX399010	JX399041	JX399074
*P. chamaeropis*	CBS 186.71	KM199326	KM199391	KM199473
*P. clavata*	MFLUCC 12-0268	JX398990	JX399025	JX399056
*P. colombiensis*	CBS 118553	KM199307	KM199421	KM199488
*P. digitalis*	ICMP 5434	KP781879	KP781883	-
*P. dilucida*	LC3232	KX894961	KX895293	KX895178
*P. diploclisiae*	CBS 115587	KM199320	KM199419	KM199486
*P. distincta*	LC8185	KY464139	KY464159	KY464149
*P. diversiseta*	MFLUCC 12-0287	JX399009	JX399040	JX399073
*P. dracontomelon*	MFUCC 10-0149	KP781877	-	KP781880
*P. ericacearum*	IFRDCC 2439	KC537807	KC537821	KC537814
*P. ericacearum*	OP023	KC537807	KC537821	KC537814
*P. formosana*	NTUCC 17-010	MH809382	MH809386	MH809390
*P. furcata*	MFLUCC 12-0054	JQ683724	JQ683708	JQ683740
*P. gaultheria*	IFRD 411-014	KC537805	KC537819	KC537812
*P. gaultheria*	OP137	KC537805	KC537819	KC537812
*P. grevilleae*	CBS 114127	KM199300	KM199407	KM199504
*P. hawaiiensis*	CBS 114491	KM199339	KM199428	KM199514
*P. hispanica*	CBS 115391	MH553981	MH554640	MH554399
*P. hollandica*	CBS 265.33	KM199328	KM199388	KM199481
*P. humus*	CBS 336.97	KM199317	KM199420	KM199484
*P. inflexa*	MFLUCC 12-0270	JX399008	JX399039	JX399072
*P. intermedia*	MFLUCC 12-0259	JX398993	JX399028	JX399059
*P. italiana*	MFLUCC 12-0657	KP781878	KP781882	KP781881
*P. jesteri*	CBS109350	KM199380	KM199468	KM199554
*P. jiangxiensis*	LC4399	KX895009	KX895341	KX895227
*P. jinchanghensis*	LC8191	KY464145	KY464165	KY464155
*P. kenyana*	CBS 442.67	KM199302	KM199395	KM199502
*P. knightiae*	CBS 114138	KM199310	KM199408	KM199497
*P. krabiensis*	MFLUCC 16-0260	MH388360	MH412722	MH388395
*P. leucadendri*	CBS 121417	MH553987	MH554654	MH554412
*P. licualacola*	HGUP 4057	KC492509	KC481683	KC481684
*P. linearis*	MFLUCC 12-0271	JX398992	JX399027	JX399058
*P. longiappendiculata*	LC3013	KX894939	KX895271	KX895156
*P. lushanensis*	LC8183	KY464137	KY464157	KY464147
*P. lushanensis*	OP086	KC537804	KC537818	KC537811
*P. macadamiae*	BRIP 63738	KX186588	KX186680	KX186621
*P. malayana*	CBS 102220	KM199306	KM199411	KM199482
*P. monochaeta*	CBS 144.97	KM199327	KM199386	KM199479
*P. montelica*	MFLUCC 12-0279	JX399012	JX399043	JX399076
*P. neolitseae*	NTUCC 17-012	MH809384	MH809388	MH809392
*P. novae-hollandiae*	CBS 130973	KM199337	KM199425	KM199511
*P. oryzae*	CBS 353.69	KM199299	KM199398	KM199496
*P. pandanicola*	MFLUCC 16-0255	MH388361	MH412723	MH388396
*P. papuana*	CBS 331.96	KM199321	KM199413	KM199491
*P. parva*	CBS 265.37	KM199312	KM199404	KM199508
*P. pinicola*	KUMCC 19-0183	MN412636	MN417507	MN417509
*P. pinicola*	KUMCC 19-0203	MN412637	MN417508	MN417510
*P. portugalica*	CBS 393.48	KM199335	KM199422	KM199510
*P. rhododendri*	IFRDCC 2399	KC537804	KC537818	KC537811
*P. rhodomyrtus*	HGUP 4230	KF412648	KC537818	KF412645
*P. rosea*	CL0441	KY228790	-	-
*P. rosea*	MFLUCC 12-0258	JX399005	JX399036	JX399069
*P. scoparia*	CBS 176.25	KM199330	KM199393	KM199478
*P. sequoia*	MFLUCC 13-0399	KX572339	-	-
*P. shorea*	MFLUCC 12-0314	KJ503811	KJ503814	KJ503817
*P. spathulata*	CBS 356.86	KM199338	KM199423	KM199513
*P. spathuliappendiculata*	CBS 144035	MH554172	MH554845	MH554607
*P. telopeae*	CBS 114161	KM199296	KM199403	KM199500
*P. terricola*	CBS 141.69	MH554004	MH554680	MH554438
*P. trachicarpicola*	IFRDCC 2240	NR_120109	-	-
*P. trachicarpicola*	OP143	JQ845947	JQ845945	JQ845946
*P. unicolor*	MFLUCC 12-0276	JX398999	JX399030	JX399063
*P. verruculosa*	MFLUCC 12-0274	JX398996	-	JX399061
*P. yanglingensis*	LC3375	KX894975	KX895307	KX895192
